# Gray Matter Morphometry Correlates with Attentional Efficiency in Young-Adult Multiple Sclerosis

**DOI:** 10.3390/brainsci11010080

**Published:** 2021-01-09

**Authors:** Sindhuja T. Govindarajan, Ruiqi Pan, Lauren Krupp, Leigh Charvet, Tim Q. Duong

**Affiliations:** 1 Department of Radiology, Stony Brook University School of Medicine, Stony Brook, NY 11794, USA; tirumals@pennmedicine.upenn.edu (S.T.G.); ruiqipanedu@gmail.com (R.P.); 2 Department of Neurology, New York University Grossman School of Medicine, New York, NY 10016, USA; Lauren.Krupp@nyulangone.org (L.K.); Leigh.Charvet@nyulangone.org (L.C.); 3 Department of Radiology, Albert Einstein College of Medicine and Montefiore Health System, Bronx, NY 10461, USA

**Keywords:** pediatric-onset multiple sclerosis, cognition, executive control, attention, atrophy, cortical thickness, frontoparietal attention network

## Abstract

Slowed processing on the alerting, orienting and executive control components of attention measured using the Attention Network Test-Interactions (ANT-I) have been widely reported in multiple sclerosis (MS). Despite the assumption that these components correspond to specific neuroanatomical networks in the brain, little is known about gray matter changes that occur in MS and their association with ANT-I performance. We investigated vertex-wise cortical thickness changes and deep gray matter volumetric changes in young MS participants (N = 21, age range: 18–35) with pediatric or young-adult onset and mild disease severity. ANT-I scores and cortical thickness were not significantly different between MS participants and healthy volunteers (N = 19, age range: 18–35), but thalamic volumes were significantly lower in MS. Slowed reaction times on the alerting component in MS correlated significantly with reduced volume of the right pallidum in MS. Slowed reaction times on executive control component correlated significantly with reduced thickness in the frontal, parietal and visual cortical areas and with reduced volume of the left putamen in MS. These findings demonstrate associations between gray matter changes and attentional performance even in the absence of widespread atrophy or slowed attentional processes.

## 1. Introduction

Multiple sclerosis (MS) is a chronic and progressive disease of the central nervous system, marked by periods of acute neuro inflammation and demyelination. Cognitive difficulties are estimated to occur in more than 30% of younger patients [1], and up to 70% of patients across the lifespan [2], often affecting occupational and daily functioning with reductions in overall quality of life [3]. Slowed information processing speed and attention are among the most common cognitive changes observed in MS [4], which in turn can negatively and broadly influence additional cognitive processes including executive functions and memory [5,6,7]. Although a majority of MS patients experience onset in their 20s and 30s [8], 3–5% of MS patients are diagnosed before 18 years of age [9,10]. Altered attention and processing speed in pediatric and young adult cohorts are particularly concerning because they can interfere with educational and occupational attainment. By definition, those with earlier onset will have a longer disease course and may be more susceptible to accumulation of disability into adulthood [11,12,13].

A multitude of studies on attentional processing in MS report slower reaction times on tasks of visuospatial attention, specifically the Attention Network Test (ANT) and its variant, the Attention Network Test-Interactions (ANT-I). The ANT and ANT-I are experimental tasks that can reliably measure the efficiency of discrete anatomical networks that perform the alerting (AE), orienting (ON) and executive control (EXE) components of attention [14]. Slowed reaction times in some or all of these components have been observed early MS [15], relapsing remitting (RR) MS [16,17,18], and primary and secondary progressive (PPMS and SPMS) [19,20] phenotypes. Despite the assumption that ANT-I measurements reflect the integrity of specific anatomical networks in MS, only two studies have tried to identify the neural correlates associated with ANT-I performance in MS. One study classified MS participants as mild or abnormal on magnetic resonance images (MRI) based on volumetric assessment of white matter (WM) lesions, ventricles and thalami [21] and reported that ANT-I scores were significantly different between mild-MRI and abnormal-MRI. The other study identified ANT-I associations with functional activity in the brain measured using electroencephalogram [22]. However, no studies have confirmed that ANT-I scores reflect changes in the corresponding neuroanatomical networks in MS. 

We hypothesized that ANTI-I, designed to measure efficiency of discrete anatomical networks, would be associated with morphometric changes in gray matter (GM) regions within the corresponding attentional networks. Cortical thinning and deep GM volume loss have been detected at disease onset and are related to worsening cognitive and physical disability in all MS sub-types [23,24,25]. The goal of this study was to evaluate vertex-wise cortical thickness and subcortical GM volume changes, and their correlation with performance on AE, ON and EXE in MS patients with a pediatric or young adult onset and mild disease severity. 

## 2. Materials and Methods

### 2.1. Study Population

The Institutional Review Board of Stony Brook University approved all study procedures (#516,105). All participants provided written informed consent to participate in this study. Young relapsing remitting MS (RRMS) participants in early adulthood (MS, *N* = 21, 12F/9M, age = 25.7 ± 5.2 years, age range = 18–35 years) with pediatric or young adult onset (age at onset = 19.7 ± 6.6 years) were recruited for this study through the Lourie Center for Pediatric MS at Stony Brook University Health Center. Healthy controls (HC) in early adulthood (HC, *N* = 19, 11F/8M, age = 22.6 ± 2.3 years, age range = 18–29 years) were also recruited using community-based advertisements from February to December 2015. Inclusion criteria for the MS sample were a diagnosis of MS, Expanded Disability Status Scale (EDSS) [26] score between 0 to 6, no relapse or steroid treatments within one month of data acquisition. All participants were fluent in the English language (having learned English prior to age 6 and not currently enrolled in an English Language Learner program), and scored in at least the average range for single word reading recognition on the Wide Range Achievement Test-3rd version (WRAT-3 [27]). Beck Depression Inventory-Fast Screen (BDI-FS [28]) was also performed and only those participants with BDI-FS score lower than 4 (not depressed) were included in the study. Participants diagnosed with psychiatric disorders, substance use disorders, neurological conditions (other than MS for patients) were excluded from the study. The cohort includes a subset of pediatric onset MS participants (*N* = 14) and HC (*N* = 8) previously reported in a diffusion tensor imaging study of white matter damage associations with visual information processing speed [29].

### 2.2. Attention Network Test-Interactions (ANT-I)

As part of a larger cognitive testing battery, participants were administered the Attentional Networks Test-Interactions (ANT-I) [14,30]. This computer-based assessment measures reaction time (RT) across blocked trials testing the alerting (AE), orienting (ON) and executive control (EXE) network components of attention. Participants are asked to indicate if the central arrow presented on the screen, flanked by two additional arrows on either side, is pointing left or right by pressing the corresponding buttons. Auditory and visual cues are presented before target stimuli and provide different information about the target. A central fixation cross was used in the no-cue condition where the participants were not warned of the upcoming target stimulus. A 50 ms audio cue alerts the participants about the upcoming target. A visual spatial cue (such as an asterisk) placed in the same location (valid cue) or in the opposite location (invalid cue) of the target orients the participants’ attention to the location of the upcoming target stimulus. Participants were presented with the central arrow flanked on either side by arrows pointing in the same direction (congruent condition), or by arrows pointing in the opposite direction (incongruent condition) to manipulate the executive control component of attention networks.

AE scores were calculated by subtracting the mean RT of audio-cue condition from the mean RT of no-cue conditions. ON scores were calculated by subtracting the mean RT of valid-cue conditions from the mean RT of invalid-cue conditions. EXE scores were calculated by subtracting the mean reaction time on the congruent conditions from the mean reaction time on the incongruent (conflicting stimuli) conditions. Higher scores on these tests would suggest poorer efficiency on the corresponding attentional networks.

### 2.3. Symbol Digits Modality Test (SDMT)

As a standard for screening for MS cognitive impairment [31], the oral condition of the SDMT, a 90 sec speeded visual information processing test was administered to all participants. Participants were presented a key with nine symbols paired with digits 1–9 and were asked to match symbols to digits on additional rows of symbols. Total correct scores were transformed to age-normative z-scores [32]. 

### 2.4. MRI Acquisition

MR images were acquired using a 20-channel head coil on a 3 Tesla Siemens Biograph mMR in Stony Brook University hospital. Structural imaging included a 3D T_1_-weighted (T_1_-w) Magnetization Prepared Rapid Acquisition Gradient Echo (MPRAGE) sequence (TR/TE = 2300/2.43 ms, 1 mm^3^ isotropic voxel resolution) and a 3D T_2_-weighted Fluid Attenuated Inversion Recovery (FLAIR) sequence (TR/TE = 5000/404 ms, 1 mm^3^ isotropic voxel size). 

### 2.5. Automatic Lesion Segmentation and Filling

WM lesion filling or in-painting was performed on T1-weighted images as it has been shown to increase accuracy of cortical thickness estimation in MS brains [33]. WM lesions were segmented on FLAIR and T1 images using the lesion prediction algorithm part of the Lesion Segmentation Toolbox [34] for Statistical Parametric Mapping (SPM12, London, UK) software package. The predicted lesion masks were then used to automatically replace hypo-intense lesion voxels to match the intensity of surrounding normal appearing WM tissue in T1-weighted images [35]. 

### 2.6. Cortical Thickness Estimation

The lesion filled structural MRI data were preprocessed using the fully automatic Computational Anatomy Toolbox (CAT12, Jena, Germany) software package, also built into SPM12. The estimation of cortical thickness in CAT is a projection-based thickness (PBT) method described in Dahnke et al. [36]. Briefly, anatomical images are segmented into GM, WM and cerebrospinal fluid (CSF), and the distance of each GM voxel from both the WM boundary and the CSF boundary are calculated. A central surface along the cortical ribbon is estimated and the local maxima (cortical thickness) is projected onto other gray matter voxels by using a neighbor relationship described by the WM distance. The projected thickness surfaces from all participants were spherically registered to the built-in template space and smoothed using 15 mm kernels before entering a vertex specific statistical model. 

### 2.7. Subcortical Segmentation

Automated subcortical segmentation was performed using multi-atlas joint label fusion (JLF) implemented as part of Advanced Normalization Tools (ANTs) [37]. JLF algorithm uses pairs of T1-w anatomical images and the corresponding manually annotated labels to create subject specific probabilistic segmentations. The knowledge-transfer from multiple atlases mitigates registration-based errors that occur in standard atlas-based segmentation techniques. In this study, 20 image and label pairs from the openly available Mindboggle-101 dataset [38] consisting of the Open access series of imaging studies (OASIS) test retest (TRT-20) images were used. Volumes of 7 bilateral subcortical regions: thalamus, caudate, putamen, pallidum, amygdala, nucleus accumbens and hippocampus were extracted for statistical analysis. Volume measures were normalized for head size using total intracranial volume (TIV) measures calculated during CAT12 segmentation for cortical thickness. 

### 2.8. Statistical Analyses

All statistical analyses were performed on the residuals of AE, ON and EXE scores after removing any effect of age. Similarly, subcortical volumes normalized for TIV were also age corrected before statistical analyses.

Group comparisons for age were performed using two-sample *t*-test with equal variance, whereas comparisons for the different test scores were performed using two-sample *t*-tests with unequal variance.

Average cortical thickness in each hemisphere was compared between HC and MS using two-sample *t*-tests. Full factorial models were created in CAT 12 for vertex-wise analysis of cortical thickness data. Vertex-wise group differences were performed using a two sample *t*-test model with age as a variable of no interest. Correlation between cortical thickness and AE, ON and EXE scores were performed using general linear models with age as a variable of no interest. All vertex-wise analyses were corrected for multiple comparisons using threshold free cluster enhancement (TFCE) implemented in the TFCE toolbox (TFCE, Jena, Germany). Clusters of significant group differences or correlations that survived TFCE corrections (at *p* < 0.05) were delineated using the Desikan-Killiany-Tourville atlas. 

Subcortical volumes extracted from JLF segmentations and normalized for head size and age were compared between groups using two-sample *t*-tests with unequal variance. Correlation between normalized volume measures and age-adjusted AE, ON, EXE scores were performed using simple linear regression analysis. Uncorrected *p*-values for significant group differences and correlations are reported, with notes on whether the effect would pass Bonferroni correction for multiple comparisons.

Correlation analyses were also performed against the morphometric measures and SDMT-z scores, the standard test for cognitive impairment.

## 3. Results

### 3.1. Demographic and Clinical Characteristics

The demographic characteristics of HC and MS samples are summarized in Table 1. MS participants were between 18 and 35 years of age, and older on average than healthy controls who were between 19 and 29 years of age (*p* < 0.05). The mean disease duration of MS participants was 5.9 ± 3.5 at the time of the study. We included those participants with mild (EDSS 0–3.5) and moderate (EDSS 4.0–6.0) neurologic disability. As would be expected with a sample early in disease, the majority of participants had mild neurologic disability (see Table 1 for distribution), with *n* = 18/21 with scores of 3.5 or less, while only n = 3/21 had moderate disability. All 21 MS participants had SDMTz; however, two MS participants did not have complete information on the ANT-I and were hence excluded in the ANT-I analyses. SDMTz was not significantly different between HC and MS (*p* = 0.17). However, 6 out of the 21 MS participants scored lower than −1.5 on SDMT-z, the clinical criterion for diagnosis of cognitive impairment. None of the HC in this cohort scored below −1.5 on SDMT-z. Reaction times on AE, ON and EXE components were not significantly different between HC and MS. 

### 3.2. Cortical Thickness Group Differences

**Figure 1:**
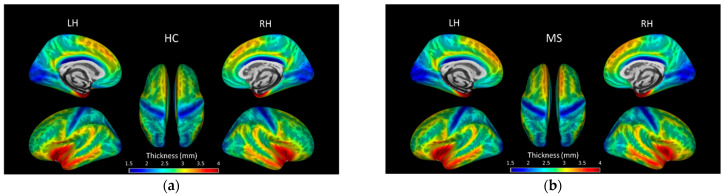
Lateral, medial and superior views of brain surface maps of mean cortical thickness (mm) in (**a**) healthy controls (HC), and (**b**) multiple sclerosis (MS) participants.

Average cortical thickness in each hemisphere was not significantly different between HC and MS (*p* > 0.05). Figure 1 shows group average cortical thickness overlaid on inflated surfaces. Vertex-wise analysis revealed significant differences in local cortical thickness between HC and MS, but the differences did not reach significance when corrected for multiple comparisons using TFCE. 

### 3.3. Subcortical GM Volume Group Differences

Table 2 shows the average volumes of subcortical GM structures normalized for TIV. Age and TIV adjusted structural volumes were significantly different between HC and MS. Specifically, MS participants had significantly lower volumes of the right caudate (*p* = 0.04), left pallidum (*p* = 0.01) and bilateral thalami (*p* = 0.002) compared to HC after correcting for age. Following Bonferroni correction (threshold *p* < 0.0036), the group differences in the bilateral thalami remained significant.

### 3.4. Vertex-Wise Cortical Thickness Correlations

Vertex-wise correlation analysis revealed no significant correlations between cortical thickness and any of the four scores in HC. In the MS group, a significant negative correlation was observed between cortical thickness and EXE after TFCE correction for multiple comparisons (*p* < 0.05). Correlation of cortical thickness and scores on SDMT, AE or ON tests did not reach significance after correction for multiple comparisons.

**Figure 2:**
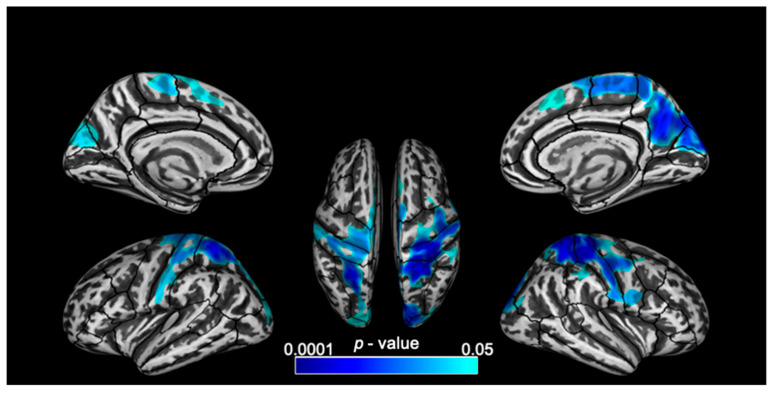
Lateral, medial and superior views of the brain with clusters of significant (*p* < 0.05, corrected for multiple comparisons) negative correlation between cortical thickness and EXE scores (from the Attention Network Tests-Interaction) overlaid on inflated surfaces.

EXE scores correlated with cortical thickness in frontal, parietal and occipital regions across both hemispheres in MS. The largest clusters of significant negative correlation were observed in the superior parietal and post central regions (over 10,000 vertices bilaterally) followed by the right precuneus. Other clusters of significant negative correlation were also observed in the precentral, paracentral, superior frontal, cuneus, lateral occipital and right supramarginal regions (Figure 2 and Table 3). 

### 3.5. Subcortical Volumes Correlations

Significant correlations were observed in MS participants between adjusted GM volumes and SDMTz, AE, and EXE (Table 4), but not between GM volumes and ON scores. No significant correlations were observed between GM volumes and any of the 4 tests in HC.

SDMTz correlated positively with the volume of left nucleus accumbens (r = 0.49, *p* = 0.024), however this correlation did not survive the Bonferroni correction for multiple comparisons (*p* < 0.00089).

AE scores correlated significantly with volumes of bilateral pallidum (LH: correlation coefficient, r = −0.57, *p* = 0.011; RH: r = −0.72, *p* = 0.0006) and bilateral thalami (LH: r = −0.59, *p* = 0.0074; RH: r = −0.51, *p* = 0.027). Only the correlation between right pallidum and AE scores survived Bonferroni correction (*p* < 0.00089) for multiple comparisons.

EXE scores correlated significantly with volumes of the right pallidum (r = −0.51, *p* = 0.025), bilateral putamen (LH: r = −0.66, *p* = 0.0021; RH: r = −0.59, *p* = 0.0073), left thalamus (r = −0.46, *p* = 0.048) and left accumbens (r = −0.52, *p* = 0.022). However, none of these correlations survived Bonferroni correction (*p* < 0.00089).

## 4. Discussion

This study investigated cortical thickness and subcortical GM volumes in a cohort of young RRMS participants and its association with attentional efficiency on specific sub-networks. We found: (i) MS participants showed no significant global or vertex-wise cortical thinning but showed reduced volume of subcortical GM structures, especially the thalamus, compared to healthy controls, (ii) slowed reaction times on the alerting component correlated with lower volume of the right pallidum in MS, (iii) slowed reaction times on the executive control component correlated with reduced thickness in the frontal, parietal and visual cortices, and lower volume of the left putamen in MS, and (iv) there were no significant associations between alerting function and cortical thickness, and between orienting function OR SDMT and either morphometric measures in MS. 

### 4.1. Morphometric Changes

There were no significant differences in global average or vertex-wise cortical thickness between groups, suggesting no cortical atrophy in this relatively mild disease cohort. Previous MS studies have reported significant thinning of cortical gray matter [24,39,40,41], likely because those participants were more advanced in their disease course, or older in age. 

Significant GM volume loss was found in our MS participants in the bilateral thalami, a known association of MS pathology. Deep GM atrophy could be a result of inflammation or demyelination, neuronal loss and Wallerian degeneration [42,43]. Our results from young and minimally impaired RRMS participants suggests that even if cortical atrophy is not widespread yet, deep GM atrophy might already be present, as reported in other studies [23].

### 4.2. Attention Network Test-Interactions

The MS participants performed similar to healthy controls on all three components of the ANT-I tests. However, it is worth noting that MS participants had a wider range of scores with the standard deviation almost twice as high in some tests compared to controls. A similar lack of significant slowing in all three components has been shown in an early MS cohort (<10 years of disease duration) [14]. Other studies investigating ANT/ANT-I scores in older or more impaired MS cohorts have found significantly slower reaction times on AE [15,16,19,22], ON [22], and EXE [17,19] components, or on an averaged ANT score [18,21].

The ANT and ANT-I are widely used to assess the attention-related processing speed in healthy subjects [14,44], aging [45] and patients with attentional deficits, including attention-deficit/hyperactivity disorder (ADHD) [46,47,48], depression [49], schizophrenia [50] and Parkinson’s disease [51]. Neuroimaging studies in healthy subjects have uncovered associations between ANT/ANT-I scores and cortical activations in task or resting state functional magnetic resonance imaging [52,53], structural connectivity from diffusion tensor imaging [54] and macroscopic brain measurements such as gray matter volume and cortical thickness from T1-weighted imaging [44].

To our knowledge, this is the first MRI study to show the correlation between attentional network tests and cortical thickness and subcortical volume changes in GM regions in an MS sample.

### 4.3. Cortical Thickness Correlation with EXE Network

Slower reaction times on the executive control component were significantly correlated with cortical thickness within the fronto-parietal attention network and parts of the visual area. Several MS studies note frontal/parietal atrophy in MS participants correlated with many cognitive deficits, including fatigue, processing speed, memory and attention [43,55,56]. Functional MRI studies investigating top-down attentional control have previously proposed a dual-network model for executive control function [57,58]. The fronto-parietal network including frontal cortices and precuneus regions provides rapid signals for executive control initiation and adaptation whereas the cingulo-opercular network is responsible for sustained stable control over the entire task epoch [58,59]. Our finding suggests that our MS participants experienced difficulties in rapid initiation and adjustment of executive control. 

### 4.4. Correlation of Subcortical GM Volumes with AE Networks

Slower reaction times on the alerting component were associated with lower volumes of the right pallidum. The pallidum, along with the putamen and the caudate nucleus form the basal ganglia, and are part of discrete, somatotopically distributed circuits essential for various functions including cognition [56]. Our finding is consistent with previous findings of moderate correlations between pallidum, putamen and thalamus volumes and executive function, working memory, attention and processing speed in MS [60,61,62]. 

Our findings suggest that in young MS with relatively mild disease severity, even in the absence of significant group differences in morphometry or attentional performance, there exists a correlation between attention network scores and the corresponding neuroanatomical networks.

### 4.5. Limitation and Future Work

Vertex-wise and global cortical thickness measures were not significantly different between the groups in our study. The lack of group differences could suggest a benign or early disease course of this MS cohort or it could be due to the smaller sample size in this study. In any case, we were able to identify vertices within functionally-relevant GM regions which correlated with cognitive performance. Structural changes such as GM lesion formation, or metabolic and physiological changes, such as changes in glucose and oxygen metabolism, blood flow or function, could precede volumetric changes detectable on T1-w MRI [63,64,65]. Future investigations with multiparametric imaging might consider these measurements. Future studies with larger samples and a wider range of disease severity can corroborate the evidence shown in our study. Additionally, longitudinal monitoring of young MS patients through accumulation of disease burden could provide valuable insights into disease trajectories and allow risk identification for cognitive decline. 

## 5. Conclusions

Cortical thickness and subcortical volume changes in MS were associated with slowed processing on specific attention network tests for alerting and executive control functions in a cohort of young onset MS participants with relatively mild disease severity. Subcortical gray matter volume atrophy and cortical thickness changes could be early markers of MS pathophysiology prior to onset of cognitive deficits. 

## Figures and Tables

**Table 1:** Demographic and clinical characteristics of healthy controls (HC) and multiple sclerosis (MS) participants.

Demographics	HC	MS	*p* Value
Number of subjects	19 (8M/11F)	21 (9M/12F)	NA
Age ± SD (years)	22.6 ± 2.3	25.7 ± 5.2 *	0.02
Age range (years)	19–29	18–35	
Age at onset ± SD (years)	-	19.7 ± 6.55	NA
Age range at onset (years)	-	10.2–34.0	NA
Disease duration ± SD (years)	-	5.9 ± 3.5	NA
Disease duration range (years)	-	1.0–11.8	NA
Expanded Disability Status Scale (EDSS)	-	Median: 2 Range: 0–6	NA
EDSS 0–2 (*n*, %)	-	16, 76	NA
EDSS 2.5–3.5 (*n*, %)	-	2, 10	NA
EDSS 4–6 (*n*, %)	-	3, 14	NA
Symbol Digits Modality Test z-score (SDMTz)	−0.15 ± 0.90	−0.65 ± 1.31	0.17
Attention Network Test-Interaction (ANT-I) scores			
Alerting (AE)	25.8 ± 25.2	35.2 ± 39.6	0.4
Orienting (ON)	49.7 ± 13.5	49.3 ± 30.9	0.96
Executive control (EXE)	88.0 ± 19.9	98.1 ± 35.5	0.3
MRI features			
Average LH cortical thickness (mm)	2.74 ± 0.1	2.73 ± 0.1	0.84
Average RH cortical thickness (mm)	2.73 ± 0.1	2.73 ± 0.1	0.9

Note: AE, ON and EXE group differences were tested on the age-adjusted scores. However, the scores presented here are the raw uncorrected scores. * *p* < 0.05; EDSS: Expanded Disability Status Scale. SD: standard deviation. LH: Left hemisphere. RH: Right hemisphere. MRI: Magnetic resonance imaging.

**Table 2:** Mean volumes of 7 subcortical gray matter structures normalized for total intracranial volume (TIV).

GM Region	Hemisphere	Normalized (Average ± SD) × 10^−3^		Uncorrected *p* Value
		HC	MS	
Amygdala	LH	0.82 ± 0.1	0.79 ± 0.09	0.36
	RH	0.79 ± 0.1	0.80 ± 0.08	0.51
Caudate	LH	2.36 ± 0.2	2.23 ± 0.22	0.26
	RH	**2.45 ± 0.2**	**2.26 ± 0.21**	**0.04**
Hippocampus	LH	2.72 ± 0.2	2.60 ± 0.20	0.07
	RH	2.82 ± 0.2	2.77 ± 0.19	0.61
Pallidum	LH	**1.09 ± 0.1**	**1.01 ± 0.09**	**0.01**
	RH	1.04 ± 0.1	0.99 ± 0.07	0.06
Putamen	LH	3.31 ± 0.2	3.19 ± 0.26	0.22
	RH	3.19 ± 0.2	3.06 ± 0.23	0.16
Thalamus	LH	**5.90 ± 0.3**	**5.36 ± 0.49**	0.0019 *
	RH	**5.75 ± 0.3**	**5.21 ± 0.50**	0.0015 *
Accumbens	LH	0.37 ± 0.1	0.35 ± 0.04	0.18
	RH	0.33 ± 0.0	0.32 ± 0.03	0.81

Note: Regions with significant (*p* < 0.05) group differences are indicated in bold. * indicates group differences that survived Bonferroni correction for multiple comparisons. LH: left hemisphere; RH: right hemisphere.

**Table 3:** Cortical regions and the number of vertices that show significant (*p* < 0.05, corrected for multiple comparisons) negative correlation between EXE scores and cortical thickness in MS.

Brain Lobe	Region Name	Number of Significant Vertices	
		LH	RH
Frontal	Superior frontal	1176	1696
	Precentral	2184	3959
	Paracentral	1176	2262
Parietal	Postcentral	5040	5938
	Superior parietal	5040	5938
	Precuneus	336	5372
	Supramarginal		848
Occipital	Cuneus	1008	1414
	Lateral occipital	840	848
Total		16,800	28,275

Note: Total number of vertices is 327,684 (whole brain). LH: left hemisphere; RH: right hemisphere.

**Table 4:** Summary of correlation between subcortical GM volumes and normalized Symbol Digits Modality Test (SDMTz) scores, and scores on the Attention Network Tests-Interaction (ANT-I) of alerting (AE) and executive control networks (EXE). Only regions with significant (*p* < 0.05) correlation with test scores are reported.

GM Region	Hemisphere	SDMTz		AE		EXE	
		R	*p*	R	*p*	R	*p*
Pallidum	LH	-	-	−0.57	0.01	-	-
	RH	-	-	−0.72	0.0006 *	−0.51	0.025
Putamen	LH	-	-	-	-	−0.66	0.002
	RH	-	-	-	-	−0.59	0.007
Thalamus	LH	-	-	−0.59	0.007	−0.46	0.048
	RH	-	-	−0.51	0.027	-	-
Accumbens	LH	0.49	0.024	-	-	−0.52	0.022
	RH	-	-	-	-	-	-

Note: Values with a ‘*’ indicate correlations that survived Bonferroni correction for multiple comparison. R: correlation coefficient; LH: left hemisphere; RH: right hemisphere.
